# Wearable Activity Trackers for Monitoring Adherence to Home Confinement During the COVID-19 Pandemic Worldwide: Data Aggregation and Analysis

**DOI:** 10.2196/19787

**Published:** 2020-06-19

**Authors:** Jean Louis Pépin, Rosa Maria Bruno, Rui-Yi Yang, Vincent Vercamer, Paul Jouhaud, Pierre Escourrou, Pierre Boutouyrie

**Affiliations:** 1 HP2 (Hypoxia and Physio-Pathologies) Laboratory Inserm (French National Institute of Health and Medical Research) U1042 University Grenoble Alpes Grenoble France; 2 EFCR (Cardiovascular and Respiratory Function) Laboratory Grenoble Alpes University Hospital Grenoble France; 3 Inserm (French National Institute of Health and Medical Research) U970 Hôpital Européen Georges Pompidou Assistance Publique Hôpitaux de Paris Paris France; 4 Université de Paris Paris France; 5 Withings Issy les Moulineaux France; 6 Hôpital Béclère Assistance Publique Hôpitaux de Paris Paris France; 7 Université Paris Saclay Paris France

**Keywords:** wearable activity trackers, pandemic, COVID-19, home confinement, lockdown, monitoring, wearables, tracking

## Abstract

**Background:**

In the context of home confinement during the coronavirus disease (COVID-19) pandemic, objective, real-time data are needed to assess populations’ adherence to home confinement to adapt policies and control measures accordingly.

**Objective:**

The aim of this study was to determine whether wearable activity trackers could provide information regarding users' adherence to home confinement policies because of their capacity for seamless and continuous monitoring of individuals’ natural activity patterns regardless of their location.

**Methods:**

We analyzed big data from individuals using activity trackers (Withings) that count the wearer’s average daily number of steps in a number of representative nations that adopted different modalities of restriction of citizens’ activities.

**Results:**

Data on the number of steps per day from over 740,000 individuals around the world were analyzed. We demonstrate the physical activity patterns in several representative countries with total, partial, or no home confinement. The decrease in steps per day in regions with strict total home confinement ranged from 25% to 54%. Partial lockdown (characterized by social distancing measures such as school closures, bar and restaurant closures, and cancellation of public meetings but without strict home confinement) does not appear to have a significant impact on people’s activity compared to the pre-pandemic period. The absolute level of physical activity under total home confinement in European countries is around twofold that in China. In some countries, such as France and Spain, physical activity started to gradually decrease even before official commitment to lockdown as a result of initial less stringent restriction orders or self-quarantine. However, physical activity began to increase again in the last 2 weeks, suggesting a decrease in compliance with confinement orders.

**Conclusions:**

Aggregate analysis of activity tracker data with the potential for daily updates can provide information regarding adherence to home confinement policies.

## Introduction

Nationwide total home confinement is the most significant measure that has been taken to prevent the spread of coronavirus disease (COVID-19) infection [1]. Currently, over 3 billion people worldwide, one-quarter of the world’s population, are confined to their homes. However, the timing and stringency of governmental decisions have been heterogeneous; some governments have imposed total lockdown, while others have required partial or no confinement. Objective, real-time measures to assess populations’ adherence to confinement are essential to adapt policies and control measures accordingly. 

We asked whether wearable activity trackers could provide this information because of their capacity for seamless and continuous monitoring of individuals’ natural activity patterns regardless of their location [2]. Data from activity trackers enable the compilation of synchronized big data resources on human behavior with high geographical and temporal resolution. In the context of the COVID-19 pandemic, activity trackers provide a valuable data set that objectively documents the time course of adherence to home confinement worldwide in response to the outbreak. 

## Methods

We analyzed data from approximately 742,000 individuals using activity trackers that count the wearer’s average daily number of steps (Withings) in a number of representative nations that adopted different modalities of restriction of citizens’ activities. We selected the nations according to a compromise between exposure to the COVID-19 pandemic, rules of lockdown, and availability of data from a large number of activity tracker users.

The pre-pandemic period was used as a reference. For each individual, we calculated the average daily number of steps between December 1, 2019, and the date of lockdown, representing the pre-pandemic period, and the average daily number of steps between the date of lockdown and the analysis time point (4 weeks for China). These data were then aggregated across countries or regions. The paired Wilcoxon nonparametric test was used to compare the number of steps per day before and during lockdown. The data presented extend to April 13, 2020, and are averaged by days and over countries or provinces/states.  

The activity tracker used in this study was a wristwatch with an embedded accelerometer that counts steps. The performance of this tracker is reported to be one of the best among available devices [3]. The activity tracker provides the most accurate measures of step count under all three important physiological conditions (ie, treadmill, over-ground, and 24-hour conditions). The same accelerometer and algorithm were used for all individuals included in the analysis. All activity tracker wearers were informed that the anonymized data collected from them could be used for research purposes, and they provided informed consent before starting to use the activity tracker. They were allowed to withdraw their consent at any time and request deletion of their individual data.

## Results

**Figure 1:**
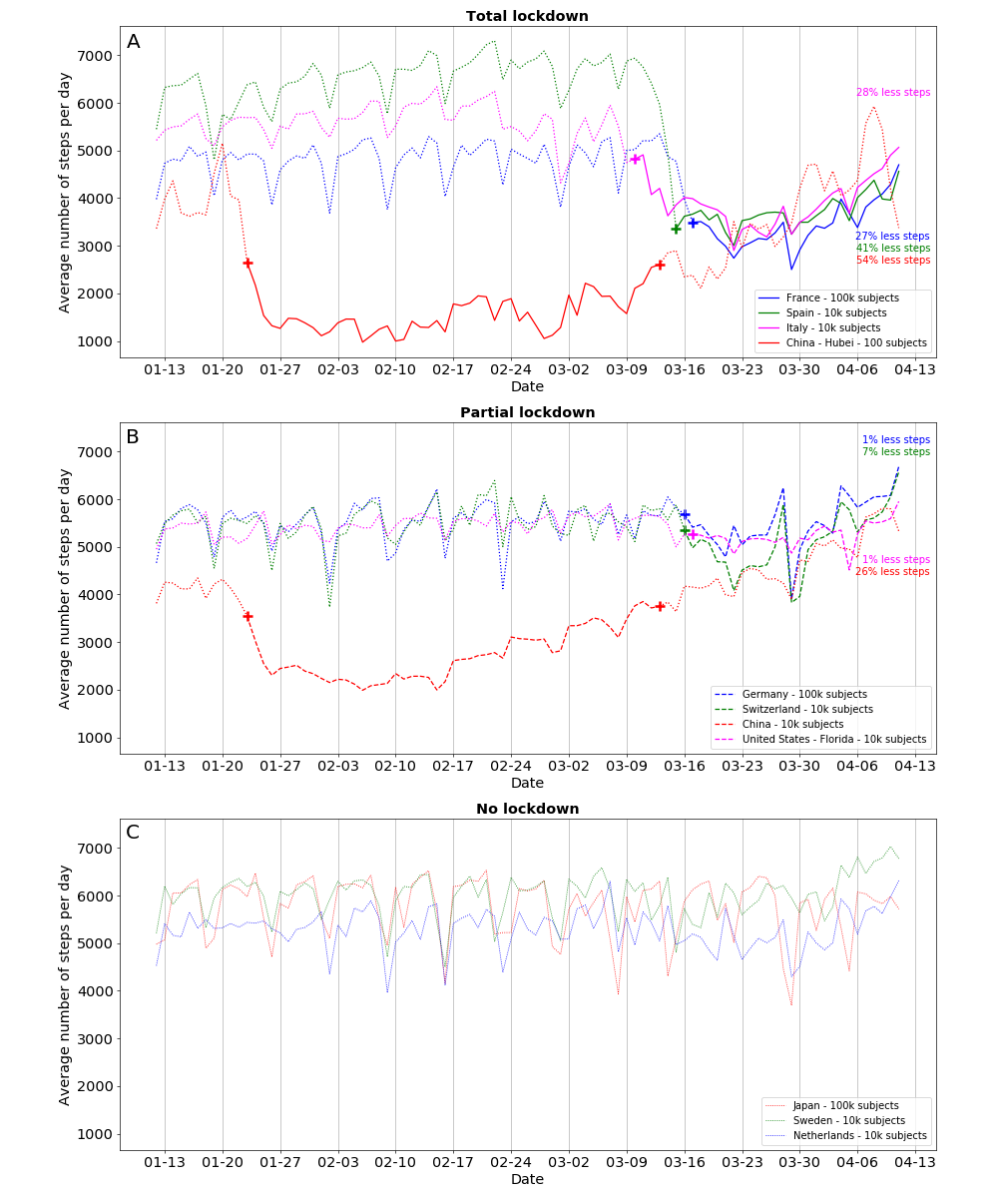
Trajectories of average daily number of steps recorded by activity trackers from January 13, 2020, to April 13, 2020, in a number of representative countries worldwide adopting total (A), partial (B), or no (C) lockdown. Solid lines indicate total lockdown periods, dashed lines indicate partial lockdown periods, and dotted lines indicate no lockdown. Crosses indicate the start and end dates of lockdown in the different countries.

Table 1 shows the number of users per country or province/state, percentage of female users, lockdown initiation dates, rules, and percentage of decrease in steps during lockdown. The most demonstrative countries are presented in Figure 1, illustrating the time course of the step count and enhancing the data in Table 1. Before the epidemic, all countries showed a stable mean number of steps per day, with periodic and reproducible decreases during weekends. In countries adopting a total lockdown, a marked decrease (from 25% to 54%) in the number of steps following the official dates of home confinement can be clearly identified (Figure 1A). Partial lockdown (characterized by social distancing measures such as school closures, bar and restaurant closures, and cancellation of public meetings but without strict home confinement) does not appear to have a significant clinical impact on people’s activity compared to the pre-pandemic period (Figure 1B), with similar activity patterns to those in nations without any restriction orders (Figure 1C). 

**Table 1:** Characteristics of the studied population.

Country	Province or state	Size of user population^a^	Proportion of women (%)^a^	Mean age (years)	Lockdown rules	Lockdown date	Baseline steps per day	Lockdown steps per day	Decrease in steps (%)	*P* value
Australia	N/A^b^	10,000	42	42	Partial	2020-03-23	5765	5302	8.0	<.001
Canada	N/A	10,000	38	43	None	N/A	5049	4708	6.8	<.001
China	N/A	10,000	19	36	Total	2020-01-23	4108	3034	26.1	<.001
China	Hubei	100	14	35	Total	2020-01-23	4375	1943	55.6	<.001
France	N/A	100,000	43	43	Total	2020-03-17	4604	3342	27.4	<.001
Germany	N/A	100,000	37	46	Partial	2020-03-16	5349	5416	–1.3	<.001
Ireland	N/A	10,000	38	42	Total	2020-03-28	5326	5356	–0.6	<.001
Italy	N/A	10,000	31	45	Total	2020-03-10	5445	3918	28.0	<.001
Italy	Lodi	100	29	45	Total	2020-02-21	5640	5035	10.7	<.001
Japan	N/A	100,000	29	43	Total	2020-04-07	5460	4581	16.1	<.001
Netherlands	N/A	10,000	38	44	None	N/A	5193	5180	0.3	<.001
Singapore	N/A	1000	33	41	None	N/A	6127	5860	4.3	<.001
Spain	N/A	10,000	36	46	Total	2020-03-15	6215	3638	41.5	<.001
Sweden	N/A	10,000	34	44	None	N/A	5681	6004	–5.7	<.001
Switzerland	N/A	10,000	40	44	Partial	2020-03-16	5325	4947	7.1	<.001
United Kingdom	N/A	100,000	39	43	Total	2020-03-23	5690	5249	7.8	<.001
United States	N/A	100,000	43	43	Partial	2020-03-22	5287	4912	7.1	<.001
United States	California	100,000	38	43	Total	2020-03-19	5508	5013	9.0	<.001
United States	Florida	10,000	44	46	Partial	2020-03-17	5303	5225	1.5	<.001
United States	Illinois	10,000	41	42	Total	2020-03-21	5415	4966	8.3	<.001
United States	New Jersey	10,000	38	43	Total	2020-03-21	5297	4693	11.4	<.001
United States	Pennsylvania	10,000	43	44	Partial	2020-03-19	5186	4974	4.1	<.001
United States	New York	10,000	39	42	Partial	2020-03-22	5776	4499	22.1	<.001
United States	Nevada	1000	42	45	Partial	2020-03-21	5391	4902	9.1	<.001

^a^The number of users having activity data on a given day is subject to variation; the numbers given in the table are representative orders of magnitude of the daily number of users having activity data.

^b^Not applicable.

## Discussion

### Principal Findings

The absolute level of physical activity under total home confinement in European countries is around twofold that in China (Table 1); this may be due to stricter governmental rules in China or different national temperaments. Interestingly, in some countries, such as France and Spain, physical activity started to gradually decrease even before official commitment to lockdown as a result of initial less stringent restriction orders or self-quarantine. However, physical activity began to increase again in the last 2 weeks, suggesting a decrease in compliance with confinement orders. Countries with partial or no lockdown policies had marginal or no changes in walking habits. Ireland was the only country with enforced confinement that showed no change in step counts. Regarding the magnitude and significance of the changes, all differences were highly significant in statistical terms (*P*<.001) because of the large number of users and because of consistent trends among users (the vast majority of users changed their step counts in the same direction, even for small changes). Clinical significance is thus arbitrary, and the weekly trends suggest spontaneous group changes (decreases on weekends). In fully locked-down countries, with the exception of Ireland, the number of steps decreased below the maximum on weekends; this shows overall good compliance with lockdown rules.

### Conclusion

Aggregate analysis of activity tracker data, with the potential for daily updates, can inform governments and stakeholders on adherence to home confinement policies as well as their efficacy without violating citizens’ privacy [4].  The data allow comparison of the effectiveness of different government policies. Finally, quantification of physical activity patterns, particularly leisure versus occupational patterns, and their consequences on cardiometabolic health are important because sport and leisure physical activities have been shown to have positive effects on cardiometabolic health, whereas occupational physical activity has not [5]. Data emerging from studies conducted during lockdown will help to address this issue.

## Abbreviations

COVID-19: coronavirus disease
